# Excluded-stomach Perforation after Roux-en-Y and One-anastomosis Gastric Bypass: A Systematic Review with Video-illustrated Case Report

**DOI:** 10.1007/s11695-026-08725-y

**Published:** 2026-05-20

**Authors:** Mohamed Hany, Ala Wafa, Mona S. Youssef, Mohamed H. Zidan, Hashem Altabbaa, Hazem Al-Momani, Dina M. Hafez, Walid El Ansari, Mohamed Samir

**Affiliations:** 1 Department of Experimental and Clinical Surgery, Medical Research Institute, Alexandria University, Alexandria, Egypt; 2 Madina Bariatric Center, Madina Women’s Hospital, Alexandria, Egypt; 3 Misurata University, Jamahiriya, Libya; 4 Alexandria University, Alexandria, Egypt; 5 Al Basheer Hospital, Amman, Jordan; 6 The Research Papyrus Lab, Alexandria, Egypt; 7 NMC Royal Khalifa Hospital, Abu Dhabi, United Arab Emirates; 8 Biostatistics Consultant, Alexandria University, Alexandria, Egypt; 9 Students Hospital, Alexandria University, Alexandria, Egypt; 10 College of Medicine, Ajman University, Ajman, United Arab Emirates

**Keywords:** Gastric remnant perforation, Excluded stomach, Roux-en-Y gastric bypass, One-anastomosis gastric bypass, Bariatric surgery complications

## Abstract

Gastric remnant or duodenal perforation after Roux-en-Y gastric bypass (RYGB) or one-anastomosis gastric bypass (OAGB) is rare and may be missed radiologically. We systematically reviewed PubMed, Scopus, and Web of Science and included 26 reports (34 patients), alongside a video-illustrated late OAGB case. Mean age was 49 years, 73.5% were female, and BMI at presentation was 33.58 kg/m²; 32 events followed RYGB and 2 OAGB, occurring a mean of 118.7 months postoperatively. CT was performed in 79.4% and more often showed free fluid than pneumoperitoneum. Laparoscopy (70.6%) with primary repair with or without omental flap or limited remnant resection achieved source control; no completion gastrectomies or 30-day deaths were reported. Early diagnostic laparoscopy should be considered in septic bypass patients with unexplained free fluid; however, the available evidence is limited to case reports and small case series, so definitive conclusions regarding incidence, comparative risk, or causative mechanisms cannot be drawn.

**Supplementary Information:**

The online version contains supplementary material available at 10.1007/s11695-026-08725-y.

## Key Points

Gastric remnant/duodenal perforation after RYGB and OAGB is rare and may present without pneumoperitoneum on CT and might present late after surgery.

CT may be non-diagnostic; free fluid without free air should maintain suspicion and prompt early diagnostic laparoscopy in persistent sepsis.

Organ-preserving surgery (primary repair ± omentoplasty or limited resection) is most reported, with no 30-day deaths or recurrences in available follow-up.

Our late OAGB case highlights the diagnostic difficulty of excluded-stomach perforation and the need for early exploration when sepsis remains unexplained.

**Supplementary Information:**

The online version contains supplementary material available at 10.1007/s11695-026-08725-y.

## Introduction

One-anastomosis gastric bypass (OAGB) is the third most commonly performed metabolic and bariatric surgery (MBS) worldwide, recognized for its technical simplicity and effective outcomes similar to Roux-en-Y gastric bypass (RYGB) [1, 2]. Like RYGB, OAGB retains a long, excluded gastric remnant that remains inaccessible to endoscopic surveillance, raising unique anatomical and physiological considerations [3].

Despite the excluded gastric remnant being seen as inert, recent studies indicate ongoing acid secretion and motility, suggesting a risk for bile reflux, mucosal injury, and, in rare cases, perforation [4, 5]. These complications are difficult to detect due to the remnants’ inaccessibility and the absence of typical radiological signs, such as pneumoperitoneum, which may be obscured by the lack of gastric aeration [6, 7].

Perforation of the excluded gastric remnant is a rare but serious complication, and its incidence and risk factors are poorly understood. Because the available literature is limited to case reports and small case series, systematic synthesis can provide descriptive and hypothesis-generating evidence but cannot establish incidence, comparative risk, or causality. To explore the available evidence, we conducted a systematic review of gastric remnant or duodenal perforation after RYGB and OAGB, examining clinical presentation, diagnosis, management, and outcomes. The accompanying video-documented OAGB case is presented as an illustrative clinical example of the diagnostic difficulty identified in the review, particularly non-diagnostic CT findings and the role of diagnostic laparoscopy for confirmation and source control.

## Methods

### Study Design

This work is a multimedia case report and systematic review of gastric remnant perforation after OAGB and RYGB, following PRISMA 2020 guidelines [8]. It aims to synthesize evidence on presentation, diagnosis, management, and outcomes. The institutional case was included as an illustrative video case to contextualize the review findings and demonstrate operative diagnosis and source control; it was not used to generate pooled estimates or infer causality. The protocol was registered on the Open Science Framework (DOI: 10.17605/OSF.IO/D4ERU) and included standard systematic review steps. A case from our institution was included with explicit informed consent for publication and use of accompanying video material.

### Search Strategy

**Fig. 1:**
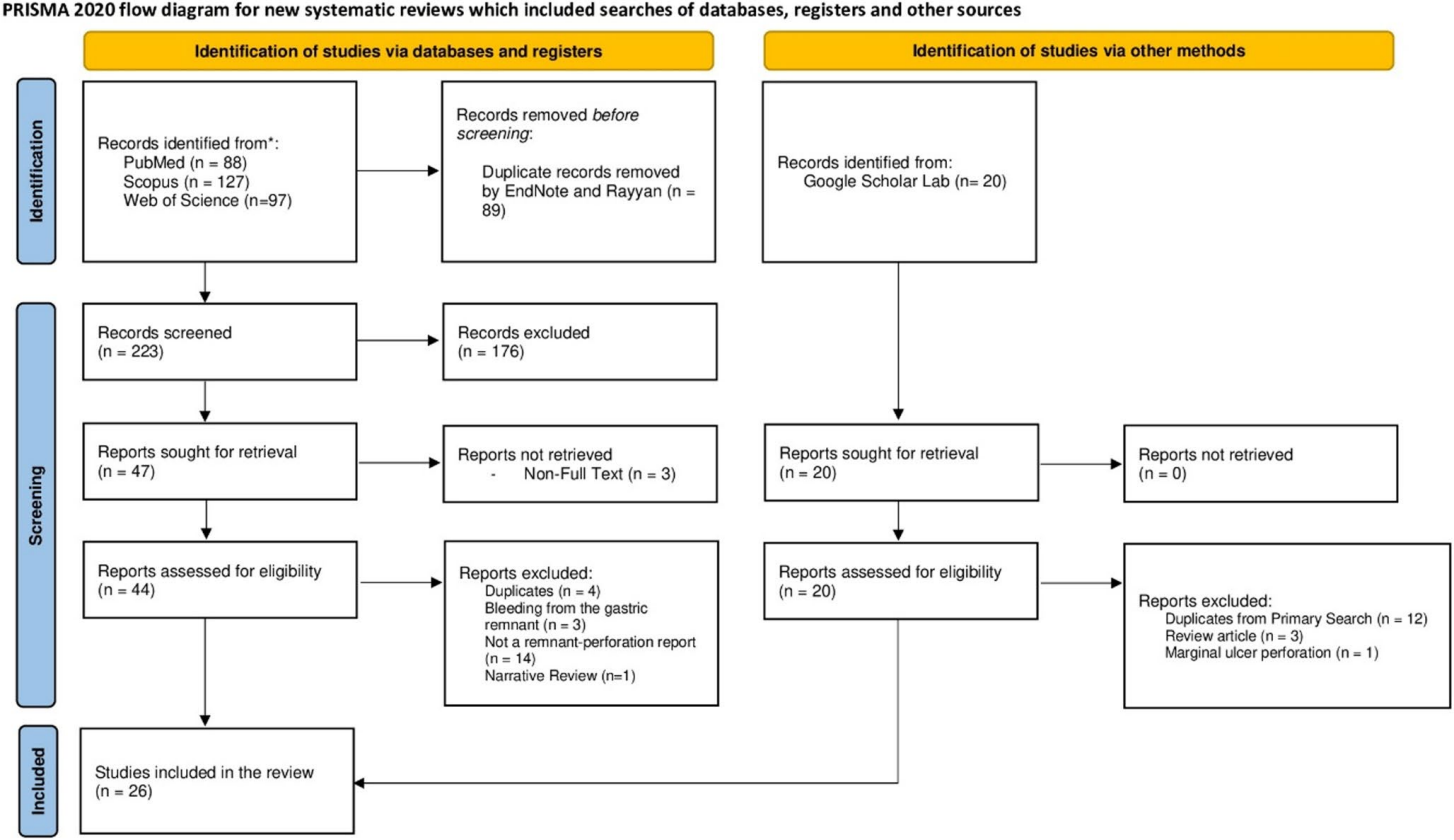
PRISMA 2020 flow diagram for study selection showing records identified across PubMed, Scopus, and Web of Science; duplicates removed in EndNote; records screened at title/abstract; full texts assessed with reasons for exclusion; and studies included in qualitative synthesis

A comprehensive search was conducted in PubMed, Scopus, and Web of Science from inception to January 2026 for reports of gastric remnant perforation after OAGB or RYGB. Search strategies are detailed in Electronic Supplementary Material 1 (ESM 1) and involved combining procedure-related terms (e.g., “OAGB,” “RYGB”) with anatomy/complication terms (e.g., “gastric remnant,” “perforation”). A supplementary manual search was performed using Google Scholar (Google Scholar Labs) to identify potentially relevant reports not captured by database indexing. Reference lists of eligible articles were also hand-searched. Records were exported to EndNote 20, where duplicates were removed before screening. The PRISMA 2020 flow diagram outlines the selection process (Fig. 1).

### Eligibility Criteria and Study Selection

Studies were eligible if they discussed gastric remnants or excluded-segment perforation after RYGB or OAGB and provided data on presentation, diagnostics, operative findings, and outcomes. Reports were excluded if they did not involve the excluded stomach/duodenum (e.g., marginal ulcers, staple-line leaks) or if they focused on other complications (hemorrhage, bezoar, and fistula without perforation). Experimental studies, technical endoscopic descriptions without new cases, and narrative reviews were also excluded. Two reviewers screened titles/abstracts in Rayyan and assessed full texts of potentially eligible articles, resolving disagreements by consensus.

### Data Charting and Extraction

Two reviewers independently extracted data using a standardized form, including author, year, country, procedure type, interval from primary surgery to perforation, suspected etiology, clinical presentation, imaging findings, intraoperative details, management, and outcomes. Helicobacter pylori (*H. Pylori*) status, relevant medications, and complications (e.g., internal hernia, marginal ischemia) were also noted when reported.

Data were entered into an Excel spreadsheet, cross-checked for accuracy, and summarized narratively and in tables to describe patterns rather than to calculate pooled estimates. Postoperative morbidity for each case was additionally graded according to the Clavien–Dindo classification of surgical complications [9], based on the level of intervention required and the occurrence of life-threatening events or mortality.

### Risk of Bias

The risk of bias of included case reports and case series was assessed using the Joanna Briggs Institute (JBI) Critical Appraisal Checklists appropriate for each design [10]. Case reports were evaluated across eight domains (demographics, history/timeline, presentation, diagnostics, intervention, post-intervention course, adverse events, and key clinical lessons), and case series across ten domains (inclusion criteria, case identification and measurement, completeness and consecutiveness of inclusion, demographic and clinical detail, outcomes/follow-up, setting/clinician description, and statistical analysis).

Each domain was rated as “yes”, “no”, or “unclear”. An overall risk-of-bias judgement was then assigned using a uniform rule-based approach: low risk (0–1 domains rated “no”/“unclear”), moderate risk (2–3 domains), and high risk (≥ 4 domains) [10].

### Statistical Analysis

All extracted variables were analyzed descriptively because the dataset consisted exclusively of case reports and small case series. Categorical variables were summarized as absolute counts and percentages. Continuous variables were inspected for distributional skewness and reported as means with standard deviations and ranges. Findings were synthesized across six analytic domains: (1) characteristics of included studies, (2) patient and event characteristics, (3) etiology and anatomical site, (4) diagnostic evaluation, (5) operative management, and (6) postoperative outcomes. No inferential statistics or meta-analytic pooling were attempted owing to heterogeneity of report types and sample size constraints.

## Results

### Study Selection

The database search identified 312 records from PubMed, Scopus, and Web of Science, and an additional 20 records through Google Scholar Labs. After the removal of 89 duplicate records using EndNote and Rayyan, 223 unique records remained for title and abstract screening. 176 records were excluded at this stage. A total of 47 reports were sought for full-text retrieval; three were not retrievable as full texts, leaving 44 articles and 20 from Google Scholar Labs assessed for eligibility. Of these, 38 were excluded for predefined reasons, and 26 publications met the inclusion criteria and were retained for qualitative synthesis, as summarized in the PRISMA 2020 flow diagram (Fig. 1) [11–36].

### Characteristics of Included Studies

Twenty-six publications met the eligibility criteria (Table 1). Twenty-two were single-patient case reports (including one editorial with an embedded case), and four was a small case series, yielding a total of 34 patients with gastric remnant perforation after metabolic–bariatric bypass. Publication years ranged from 2003 to 2025, and reports originated from Europe, the Middle East/Asia, and North America, reflecting geographically dispersed experience.

**Table 1:** Characteristics of the included studies describing gastric remnant or duodenal perforation after Roux-en-Y gastric bypass (RYGB) or one-anastomosis gastric bypass (OAGB)

Author	Year	Journal	Country	Study Type	Procedure Types Included	Number of Cases reported (*n*)	Number of Cases Included in this review (*n*)	Primary Focus
Tuero et al. [11]	2021	World Journal of Laparoscopic Surgery	Spain	Case report	RYGB	1	1	Perforation
AlZarooni et al. [12]	2020	Obesity Surgery	United Arab Emirates	Case report	OAGB	1	1	Perforation
Ebrahimi et al. [13]	2019	Annals of the Royal College of Surgeons of England	Iran	Case report	RYGB	1	1	Perforation
Suri et al. [14]	2019	Clinical Imaging	USA	Case report	RYGB	1	1	Perforation
Iranmanesh et al. [15]	2020	Obesity Surgery	USA	Case series	RYGB	7	3	Mixed (bleeding, perforation, necrosis)*
Munhoz et al. [16]	2024	EDM Case Reports	Portugal	Case report	RYGB	1	1	Perforation
Dai et al. [17]	2018	BMJ Case Reports	USA	Case report	RYGB	1	1	Perforation
Marquez et al. [18]	2023	International Journal of Surgery Case Reports	USA	Case report	RYGB	1	1	Perforation
Gnägi et al. [19]	2021	International Journal of Surgery Case Reports	Switzerland	Case report	RYGB	1	1	Perforation
Laverty et al. [20]	2021	The American Surgeon	USA	Case report	RYGB	1	1	Perforation
Papasavas et al. [21]	2003	Obesity Surgery	USA	Case report	RYGB	1	1	Perforation
Ovaere et al. [22]	2016	BMJ Case Reports	Netherlands	Case report	RYGB	1	1	Perforation
Shahabi et al. [23]	2025	Obesity Surgery	Iran	Editorial with case report	OAGB	1	1	Perforation
Williams et al. [24]	2024	Cureus	USA	Case report	RYGB	1	1	Perforation
Pereira et al. [25]	2025	Cureus	Portugal	Case report	RYGB	1	1	Perforation
Aftab et al. [26]	2025	Journal of Surgical Case Reports	USA	Case report	RYGB	1	1	Perforation (duodenal, excluded segment)
Ay et al. [27]	2022	European Research Journal	Turkey	Case report	RYGB	1	1	Perforation
Gonçalves et al. [28]	2025	Cureus	Portugal	Case report	RYGB	1	1	Perforation
Iskandar et al. [34]	2015	Case Reports in Surgery	USA	Case series	RYGB	2	2	Perforation
Mittermair et al. [29]	2007	Obesity Surgery	Austria	Case report	RYGB	1	1	Perforation
Gypen et al. [30]	2008	Obesity Surgery	Belgium	Case report	RYGB	1	1	Perforation
Nowotny et al. [31]	2020	The American Surgeon	USA	Case report	RYGB	1	1	Perforation
Peetermans et al. [32]	2019	Acta Chirurgica Belgica	Belgium	Case report	RYGB	1	1	Perforation
Pohl et al. [35]	2018	American Journal of Emergency Medicine	Switzerland	Case series	RYGB	2	2	Perforation
Zagzag et al. [36]	2018	Obesity Surgery	USA	Case series	RYGB	5	5	Perforation
Hughes et al. [33]	2023	Cureus	USA	Case report	RYGB	1	1	Perforation

n, number of patients with gastric remnant or duodenal perforation included from each report

* Mixed-focus series; only cases with gastric remnants or duodenal perforation were extracted for this review, whereas non-perforation events (for example, isolated bleeding or necrosis without perforation) were excluded from the analysis

At the patient level, 32 of 34 cases (94.1%) occurred after RYGB, whereas 2 of 34 cases (5.9%) followed OAGB. At the study level, 24 of 26 publications (92.3%) reported perforations exclusively after RYGB, and 2 of 26 publications (7.7%) involved OAGB. All included reports provided sufficient patient-level detail to extract clinical presentation, diagnostic evaluation, operative findings, and postoperative outcomes. In the mixed-focus case series, only patients with gastric remnants or duodenal perforation were abstracted for analysis (Table 1).

### Clinical and Event Characteristics

Across the 34 included patients, the mean age was 49.03 ± 13.43 years (range 22–75), and two-thirds were female. BMI at presentation averaged 33.58 ± 6.58 kg/m² (range 24–47). A broad spectrum of comorbidities and obesity-related diseases was reported, most appearing in single individuals, including systemic lupus erythematosus, hypothyroidism, prior peptic ulcer disease, hiatal hernia, renal-transplant immunosuppression, and other chronic conditions. Potential risk exposures included non-steroidal anti-inflammatory drug (NSAIDs) use in eight patients (23.5%), and isolated reports of steroid therapy, smoking, and alcohol use. *H. pylori* status was reported in a minority of cases, with mostly negative results (47.1%), one positive serology (2.9%), and one positive stool Ag (2.9%). The interval from the primary bypass to perforation was highly variable, with a mean of 118.66 ± 103.93 months (range 0.17–480), spanning very early postoperative events to perforations occurring many years after surgery (Table 2).

**Table 2:** Baseline Characteristics and Timing of Presentation Among Patients

	Overall (*N* = 34)
Age, years	
mean ± SD	49.03 ± 13.43
Range	22–75
Sex	
Male	9 (26.5)
Female	25 (73.5)
BMI at event	
mean ± SD	33.58 ± 6.58
Range	24–47
Obesity-related diseases and other comorbidities*	
Systemic lupus erythematosus	1 (2.9)
Hypothyroidism	1 (2.9)
Treated PUD	2 (5.9)
Hiatal hernia	2 (5.9)
Fatty liver	1 (2.9)
Gallbladder sludge	1 (2.9)
C-sections	1 (2.9)
Hypertension	3 (8.8)
Cholelithiasis on ultrasound	1 (2.9)
Renal transplant	1 (2.9)
Tacrolimus immunosuppression	1 (2.9)
History of reflux	1 (2.9)
Prior RYGB	1 (2.9)
Migraines	1 (2.9)
Chronic peptic ulcer disease	1 (2.9)
Degenerative joint disease	1 (2.9)
Anxiety	1 (2.9)
PTSD	1 (2.9)
Iron deficiency anemia	2 (5.9)
Diabetes	2 (5.9)
GERD	1 (2.9)
Cholecystectomy	2 (5.9)
Risk factors	
NSAIDs	8 (23.5)
Steroids	1 (2.9)
Smoking	3 (8.8)
Alcohol	4 (11.8)
Helicobacter status	
Negative	16 (47.1)
Serology Positive	1 (2.9)
Stool Ag Positive Immunosuppressed State	1 (2.9) 1 (2.9)
Time From Bypass to Event (months)	
mean ± SD	118.66 ± 103.93
Range	0.17–480

* Multiple answers were allowed

Ulcerative pathology was the predominant mechanism, accounting for 88.2% of perforations, with isolated cases attributed to ischemic or obstructive processes. Perforation sites were heterogeneous and distributed throughout the remnant stomach and duodenum. The duodenal region was the most frequently involved location (47.1%), followed by pre-pyloric perforations (11.8%), antrum (5.9%), pylorus (5.9%), and single reports involving the fundus, lesser and greater curvature, and posterior gastric wall. Reported perforation size ranged from pinpoint defects to large necrotic gaps (4–100 mm) with an average of 16.0 ± 24.78 mm, reflecting the spectrum from focal ulcer perforation to more extensive remnant destruction (Table 3).

**Table 3:** Etiology and Anatomical Site of Gastric Remnant Perforations

	Overall (*N* = 34)
Etiology category	
Ulcer	30 (88.2)
Ischemia	3 (8.8)
Obstruction	1 (2.9)
Perforation site	
Pre-pyloric	4 (11.8)
Fundus	1 (2.9)
Near staple line (medial border)	1 (2.9)
Lesser curvature	1 (2.9)
Duodenum	16 (47.1)
Gastric antrum	2 (5.9)
Pylorus	2 (5.9)
Greater curvature	1 (2.9)
Excluded remnant stomach	1 (2.9)
Gastric remnant (anterior wall)	2 (5.9)
Perforation size, mm	
mean ± SD	16.0 ± 24.78
Range	4–100

### Diagnostic Workup, Operative Management, and Post-operative Outcomes

Most patients were hemodynamically stable at presentation (67.6%), although a minority were unstable or septic (11.8%); the remainder were not explicitly classified. Leukocytosis was present in 20.6% of cases, and elevated C-reactive protein (CRP) in 14.7%. Contrast-enhanced computed tomography (CT) was performed in 27 cases (79.4%). Free intraperitoneal air was identified in only a small subset (17.6%), whereas free fluid was more common (67.6%). Additional CT findings included remnant distension (20.6%), mural thickening (26.5%), and, in some cases, features suggestive of internal hernia (8.8%). Plain radiography and abdominal ultrasonography were used selectively. Endoscopic evaluation, via standard esophagogastroduodenoscopy or double-balloon enterostomy, was performed in 29.4% of cases, and image-guided percutaneous drainage was utilized in 5.9% (Table 4).

**Table 4:** Diagnostic Evaluation and Imaging Findings in Reported Cases

	Overall (*N* = 34)
Hemodynamic Status at Presentation	
Stable	23 (67.6)
Unstable	4 (11.8)
White Cell Count	
Normal	14 (41.2)
Leukocytosis	7 (20.6)
C – Reactive Protein Level	
Normal	4 (11.8)
Positive	5 (14.7)
CT scan performed*	27 (79.4)
Free Air	6 (17.6)
Free Fluid	23 (67.6)
Remnant Distension	7 (20.6)
Wall Thickening	9 (26.5)
Internal Hernia Signs	3 (8.8)
Other Imaging*	
X-ray Abdomen/Chest	7 (20.6)
Abdominal ultrasound	7 (20.6)
CTA	1 (2.9)
Repeat CT	4 (11.8)
Other Therapeutic Methods Performed	10 (29.4)
Endoscopic Access Method	
Double Balloon Enterostomy	2 (20)
Esophagogastroduodenoscopy	8 (80)
Interventional Radiology	2 (5.9)

* Multiple answers were allowed

Laparoscopic surgery was the predominant operative modality, performed in 24 cases (70.6%), with open surgery used in 10 cases (29.4%). Primary repair was undertaken in 28 patients (82.4%), and omentoplasty was performed in 26 cases (76.5%). Stapled wedge resection of the remnant was completed in 1 case (2.9%), and partial remnant gastrectomy in 6 cases (17.6%); no patient required a completion gastrectomy. Obstruction due to an internal hernia was identified in 2 cases (5.9%), and obstruction due to a trocar-site hernia in 1 case (2.9%) (Table 5).

**Table 5:** Surgical Approaches and Repair Techniques Utilized

	Overall (*N* = 34)
Operative Approach	
Laparoscopic	24 (70.6)
Open	10 (29.4)
Procedure	
Primary Repair of ulcer defect	28 (82.4)
Primary repair with Omentoplasty	26 (76.5)
Primary repair only	2 (5.9)
Partial Remnant Gastrectomy	6 (17.6)
Stapled Wedge	1 (2.9)
Completion Gastrectomy	0 (0)
Pyloroplasty	1 (2.9)
Duodenal repair	16 (47.1)
Hernia Defect	
Internal Hernia Causing Obstruction	2 (5.9)
Petersen	1 (2.9)
mesenteric gap at JJ	1 (2.9)
Trocar Site Hernia Causing Obstruction	1 (2.9)

* Multiple answers were allowed

Postoperative proton-pump inhibitor (PPI) therapy was prescribed in 10 cases (29.4%), with lifelong therapy documented in 5 patients. Admission to the intensive care unit (ICU) was required in 4 cases. Length of hospital stay averaged 6.08 ± 3.84 days (range 3–21) (Table 6). According to the Clavien–Dindo classification, 31 patients (91.2%) experienced grade IIIb complications and 3 (8.8%) grade IV complications. No 30-day mortality was reported. Mean follow-up was 8.13 ± 12.68 months (range 0.5–38), with one patient (2.9%) recurrence; one patient (2.9%) required reoperation (Table 6).

**Table 6:** Clinical Outcomes, Postoperative Management, and Follow-up

	Overall (*N* = 34)
Postoperative PPI prescription	10 (29.4%)
lifelong PPI	5 (14.7)
No specified duration	5 (14.7)
ICU Required	4 (11.8)
Length of Hospital Stay (days)	
mean ± SD	6.08 ± 3.84
Range	3–21
Clavien-Dindo classification	
IIIB	31 (91.2)
IV	3 (8.8)
Mortality 30 days	0 (0)
Follow-up Duration (months)	
mean ± SD	8.13 ± 12.68
Range	0.5–38
Recurrence	1 (2.9)
Reoperation	1 (2.9)

* Multiple answers were allowed

### Risk of Bias Assessment

**Fig. 2:**
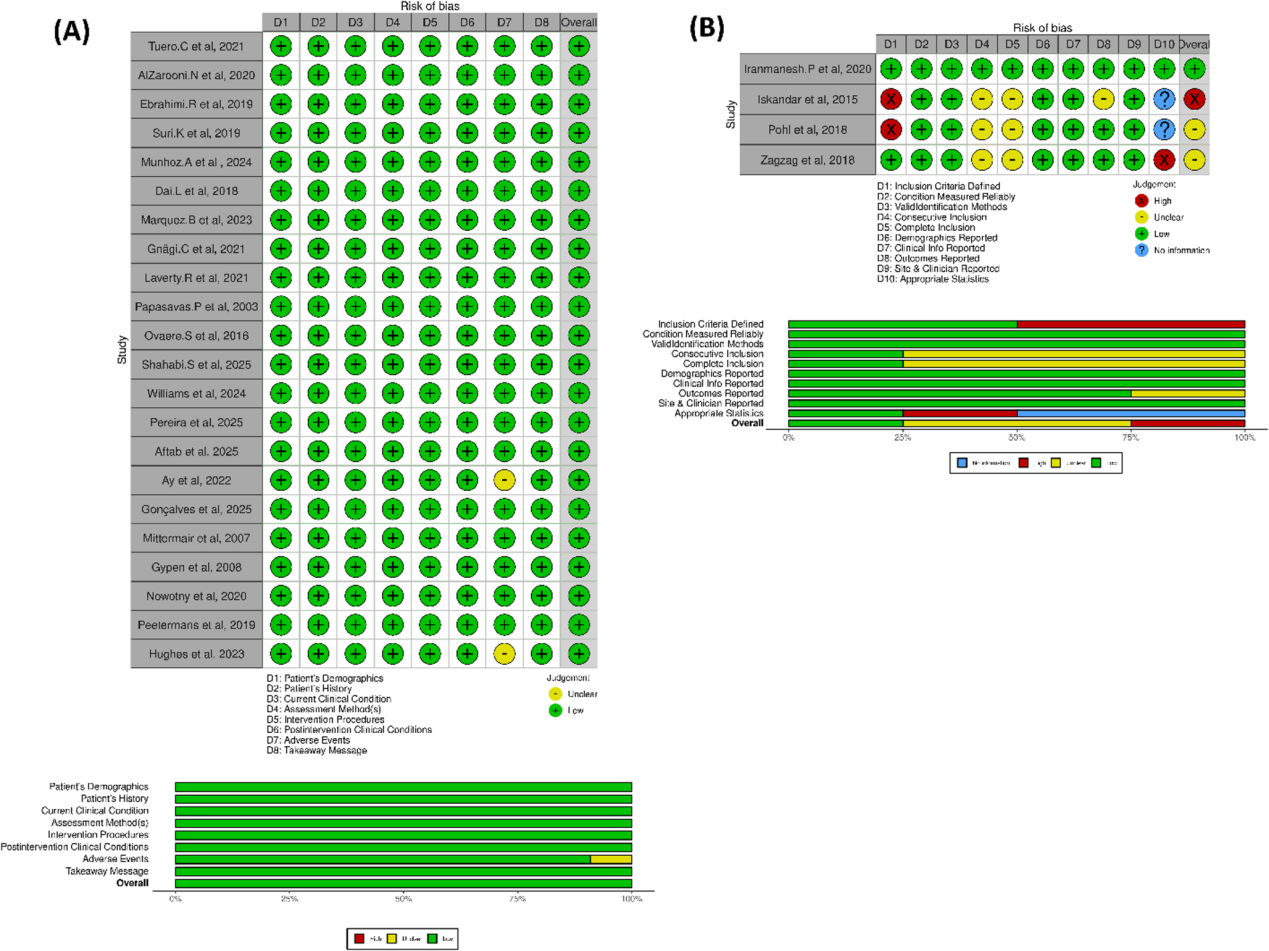
Risk-of-bias appraisal using JBI tools showing (**A**) case reports with item-level and overall judgments across eight domains (D1–D8: demographics; history/timeline; current clinical condition; assessment methods/results; intervention/procedures; post-intervention condition; adverse events; takeaway/lessons) and (**B**) the case series with judgments across ten domains (D1–D10: inclusion criteria; condition measured reliably; valid identification methods; consecutive inclusion; complete inclusion; demographics; clinical information; outcomes/follow-up; site/clinician described; appropriate statistics). Traffic-light key: green = low risk; yellow = unclear; red = high. Full checklists are provided in ESM 2

Across the included case reports, all eight JBI domains were rated low risk for every study except the Adverse Events domain (D7), which was judged unclear for Ay et al., 2022 [27] and Hughes et al., 2023 [33] because complications were not explicitly reported, overall, the case-report evidence was therefore judged predominantly low risk (Fig. 2A). For case series, risk-of-bias assessments were more variable: Iranmanesh et al., 2020 [15] met al.l ten domains with an overall low-risk judgement, whereas Iskandar et al., 2015 [34] was rated high risk overall (notably due to unclear/absent inclusion criteria and incomplete reporting), and Pohl et al., 2018 [35] and Zagzag et al., 2018 [36] were judged unclear overall, mainly driven by uncertain consecutive/complete inclusion and limited or missing statistical reporting (Fig. 2B). Study-level checklists provided in ESM 2 (Tables S2.1–S2.2).

JBI risk-of-bias judgments should not be equated with overall evidence certainty. While some reports met JBI standards, the evidence is limited due to its case-based nature, lack of denominator data, absence of comparator groups, and inability to estimate incidence or causality. Thus, findings should be seen as descriptive and hypothesis-generating only.

### Case Presentation

In January 2025, a 59-year-old female presented with severe epigastric pain radiating to the back for 12 h, accompanied by nausea, fever, and left shoulder pain, without hemodynamic compromise. Her surgical history included open cholecystectomy 17 years earlier and OAGB for severe obesity nine years earlier, after which her BMI decreased from 45 to 28 kg/m². Over the preceding year, she experienced weight regain, with a current BMI of 30 kg/m². She denied recent NSAID use and had no history of peptic ulcer disease, alcohol intake, or tobacco use.

On examination, she was febrile at 37.8 °C, tachycardic, and tachypneic, with mild abdominal distension, generalized tenderness, and shifting dullness. Laboratory tests showed leukocytosis (14,200/µL), elevated CRP (92 mg/L), markedly elevated serum amylase (1,120 U/L) and lipase (1,460 U/L), normal liver enzymes, and mild renal impairment. Abdominal ultrasound revealed moderate free fluid and dilated small bowel loops. Contrast-enhanced CT confirmed free fluid and subtle peripancreatic fat stranding, with no pneumoperitoneum, bowel obstruction, or definite gastrointestinal perforation.

Upper EGD showed no marginal ulcer, and both afferent and efferent limbs were patent. The gastric pouch contained bile and showed moderate erythematous gastritis. Radiologically guided aspiration of the intraperitoneal fluid revealed markedly elevated amylase (5,870 U/L) and lipase (6,320 U/L), raising concern for pancreatitis or occult gastrointestinal perforation. Culture grew MRSA, prompting antibiotic adjustment. A nasojejunal tube was inserted for enteral nutrition with parenteral supplementation, and PPI infusion therapy was started.

Despite these measures, the patient deteriorated with acute kidney injury requiring dialysis and ICU admission. A pigtail catheter was inserted and drained 7 L of serosanguinous fluid. Follow-up CT with oral contrast showed proximal thinning of the excluded stomach but no free air. Because of progressive renal dysfunction and increasingly turbid brownish pigtail drainage, diagnostic laparoscopy was performed on day 12, revealing serosanguinous fluid mixed with pus and an omental phlegmon.

During adhesiolysis, a 2-cm perforation was identified in the posterior wall of the excluded gastric remnant, with active bilious leakage and surrounding inflamed necrotic tissue. The gastrojejunostomy, alimentary limb, and biliopancreatic limb were intact, with no internal hernia, downstream obstruction, or other intra-abdominal pathology. The necrotic portion of the remnant was resected using a Covidien Endo GIA™ Universal Stapler, followed by extensive peritoneal lavage and placement of two drains.

The patient recovered well, remained in the ICU for two days on PPI infusion, and resumed oral intake on postoperative day 3. CT on day 7 was reassuring, and the pigtail and surgical drains were removed. She was discharged on day 10 with oral PPI therapy for six months. At the six-month follow-up, she remained asymptomatic without recurrence. Histopathological examination showed focal mucosal ulceration with acute and chronic inflammation, with no malignancy and negative staining for H. pylori.

## Discussion

This review maps the limited case-based literature on gastric remnant and duodenal perforation after RYGB and OAGB. The principal signal is not a definable incidence rate or a proven mechanism, but a recurring clinical pattern: excluded-segment perforation may occur late, may present with non-specific sepsis or abdominal pain, and may not show pneumoperitoneum on CT. In this context, the main clinical implication is the need for a high index of suspicion and early consideration of diagnostic laparoscopy when clinical deterioration persists despite non-diagnostic imaging. The accompanying OAGB case is presented to illustrate this diagnostic pathway and the role of laparoscopic source control.

### Mechanisms and Risk Factors for Remnant Perforation

Across pooled cases, perforation of the excluded stomach or duodenum appears rarely idiopathic and more commonly reflects converging ulcerogenic, chemical, and mechanical influences [4, 5, 37]. Ulcerative pathology predominated, with ischemic or obstructive causes reported only sporadically. Many patients had at least one established peptic risk factor, most often NSAID exposure, and less frequently steroids, smoking, alcohol use, or prior peptic ulcer disease, while systemic comorbidities and immunosuppression were uncommon. These factors plausibly lower the threshold for mucosal breakdown in a remnant that remains physiologically active after bypass.

Emerging evidence contradicts the assumption that the excluded stomach becomes quiescent following RYGB or OAGB. Functional and scintigraphy studies demonstrate persistent acid secretion, motility, and bile stasis years after surgery [4, 5]. Duodenal biliary and pancreatic secretions can predispose to biliary reflux into the blind remnant stomach segment, causing chronic irritation and eventual ulceration or perforation years after surgery, in an endoscopically inaccessible segment [37]. More broadly, MBS procedures induce long-lasting alterations in gastrointestinal motility, intraluminal pH, bile acid exposure, and gut hormone profiles, such that the remnant remains a dynamic, chemically active segment rather than a “switched-off” organ [38, 39]. Scintigraphic and endoscopic studies have demonstrated duodenogastric bile reflux into the excluded stomach after RYGB, with tracer or bile pooling in the remnant and histologic evidence of bile reflux gastritis in some patients [40, 41]. Similarly, multiple series and reviews have reported that bile reflux is relatively frequent after OAGB compared with other MBS procedures, with a proportion of patients developing symptomatic duodenogastro-oesophageal reflux that occasionally requires revisional surgery [42, 43]. A recent study specifically evaluating bile reflux after OAGB found that prior cholecystectomy was associated with increased scintigraphy evidence of bile reflux into the gastric remnant, reinforcing the concept that altered biliary dynamics can augment chronic chemical injury in this segment [44].

The proximal remnant location in our case is unusual for a typical distal peptic ulcer perforation and therefore warrants caution in etiological interpretation. The ulceration and inflammatory changes observed in the resected remnant raise the possibility of chronic remnant mucosal injury; however, these findings are non-specific and do not identify bile reflux, acid secretion, ischemia, transient obstruction, or any other pathway as the definitive cause. Persistent exposure to bile and pancreatic secretions after OAGB may be one potential contributor, but this remains hypothetical. Mechanical factors, such as internal hernia, downstream obstruction, or intermittent elevation of remnant pressure, may also contribute [12, 23]. Overall, the available evidence supports considering a multifactorial model in which acid secretion, bile and pancreatic reflux, systemic comorbidities, medication exposure, and mechanical factors may interact in individual patients [37]. Given the limited data from case reports and small case series, no dominant mechanism can be confirmed, and population-level risk cannot be determined.

### Diagnostic Challenges in the Excluded Stomach

From a diagnostic standpoint, excluded-stomach perforation may not behave like a conventional hollow-viscus perforation. Clinical findings can be nonspecific, and laboratory abnormalities may be absent or incomplete, making early recognition difficult in post-RYGB or post-OAGB patients.

CT should therefore not be regarded as a definitive rule-out test. In altered bypass anatomy, the excluded stomach and duodenum may contain little intraluminal gas, so perforation may present with free fluid, inflammatory change, remnant distension, or mural thickening rather than obvious pneumoperitoneum [6, 7]. Evidence of air on CT may suggest gastro-gastric fistula or distal obstruction to the biliary limb, rather than isolated remnant perforation [45]. Endoscopic assessment of the remnant or duodenum may be possible with device-assisted techniques in selected settings, but it is rarely practical in the acute setting.

In this context, persistent abdominal pain, systemic inflammatory response, or unexplained free intraperitoneal fluid after RYGB or OAGB should maintain suspicion for excluded-segment pathology, even when CT is non-diagnostic. Early diagnostic laparoscopy should be considered after initial resuscitation and reasonable exclusion of alternative diagnoses, rather than being delayed until repeated imaging confirms a clear perforation site.

### Operative Strategies and the Remnant Gastrectomy Debate

The operative literature supports a pragmatic source-control approach rather than routine radical resection. In the published reports, laparoscopic exploration, primary repair with omentoplasty, and limited remnant resection were commonly used according to local tissue quality and intraoperative findings. This is consistent with single-centre experience in acute gastric remnant complications after RYGB, where emergency operative management, most often laparoscopic, is emphasized once remnant pathology is suspected [15]. Our case follows this approach, as laparoscopic wedge resection was selected because of necrotic tissue surrounding the posterior remnant perforation.

In contrast, the role of more radical procedures such as completion remnant gastrectomy remains controversial. A recent systematic review on remnant gastrectomy with or after gastric bypass reported that resectional gastric bypass has mainly been used for selected indications, including high-risk remnant pathology or preventive resection in settings of remnant neoplasia risk [37]. In contrast, delayed remnant gastrectomy has been used for complications such as gastrogastric fistula, retrograde bile reflux gastritis, and cancer [7, 37]. Therefore, remnant resection may be appropriate in selected patients, but current evidence does not support routine prophylactic remnant gastrectomy during primary RYGB or OAGB solely to prevent rare remnant perforation [37]. At the same time, data on RYGB reversal and other major reconfiguration procedures show non-trivial morbidity, including anastomotic leaks, sepsis, and de novo reflux, underscoring that aggressive remnant surgery carries its own risks and should not be undertaken lightly [46–48].

Against this backdrop, the extremely low number of documented remnant perforations, the high success rate of primary repair or limited resection, and the absence of short-term mortality in our pooled cohort argue against routine completion gastrectomy as a preventive measure for otherwise uncomplicated RYGB or OAGB. Current evidence instead supports a pragmatic approach: careful intraoperative assessment of tissue viability and contamination, primary repair with omentoplasty when feasible, escalation to partial remnant resection when local necrosis or extensive ulceration is present, and reserving completion gastrectomy for exceptional scenarios such as diffuse, non-reconstructable disease or concurrent oncologic indications. This strategy consists of contemporary guidelines on the management of acute abdomen after MBS, which prioritize timely exploration and source control over extensive prophylactic resections in the emergency setting [49].

### Relationship between the Present Case and the Published Series

When placed in the context of the 34 patients identified in our systematic review, the present OAGB case should be interpreted as an illustrative video case rather than independent inferential evidence. Its main value is to demonstrate the clinical problem highlighted by the review: persistent sepsis and free intraperitoneal fluid despite non-diagnostic CT, with definitive diagnosis and source control achieved only at laparoscopy. Demographically, our patient falls within the dominant profile of middle-aged individuals with persistent class I–II obesity at the time of perforation, and the clinical presentation with progressive abdominal pain, sepsis, and nonspecific inflammatory markers mirrors the often-blunted symptomatology described in the pooled cohort. Similarly, CT demonstrated free intraperitoneal fluid without convincing pneumoperitoneum, consistent with the limited sensitivity of standard imaging for excluded-stomach perforation and the frequent reliance on operative exploration for definitive diagnosis.

At the same time, this case illustrates the end of the risk profile in terms of both timing and apparent aetiology. The perforation occurred almost a decade after OAGB, placing it toward the late tail of the reported temporal distribution and later than the two previously described OAGB remnant perforations, which arose within the first few postoperative years [12, 23]. Unlike many of the published patients, our case lacked conventional ulcer risk factors: there was no history of NSAID or steroid use, smoking, or alcohol excess, and Helicobacter pylori testing was negative. Histopathology of the resected remnant demonstrated multiple chronic ulcerations rather than a single focal defect, supporting a model of long-standing chemical injury, likely bile-related, superimposed on otherwise “low-risk” mucosa. This contrasts with several RYGB cases in the series where acute triggers such as medication exposure or immunosuppression were more clearly implicated.

Operatively, the management strategy in our case, early laparoscopic exploration once conservative measures had failed, with thorough adhesiolysis, identification of a posterior-wall perforation, wedge resection of the affected segment, and drain placement, aligns closely with the dominant approaches reported in the literature. The postoperative course, classified as a Clavien–Dindo grade IIIb complication due to the need for reoperation but followed by uneventful recovery and no recurrence, is also representative of the overall pattern of high morbidity by definition but low mortality and good medium-term outcomes. Collectively, these similarities and differences suggest that remnant perforation after OAGB can occur even in the absence of classic risk factors, at very late time points, and with imaging that remains equivocal.

### Risk of Bias and Limitations

The methodological quality of the included reports was generally acceptable when appraised using the JBI critical appraisal tools; most case reports and the single case series fulfilled the majority of core items, particularly concerning a clear description of clinical history, diagnostic workup, operative details, and short-term outcomes. However, several recurring weaknesses were apparent. Important variables such as *H. Pylori* status, medication exposures, and detailed comorbidity profiles were often incompletely reported or omitted entirely. Long-term follow-up was inconsistently documented, and only a minority of authors explicitly discussed potential alternative explanations for the perforation. As a consequence, although many studies were rated as low risk of bias by JBI criteria, the overall certainty of the evidence remains constrained by selective and heterogeneous reporting.

These limitations are compounded by the intrinsic constraints of the underlying study designs. All available data derive from isolated case reports and a single small series, which are inherently prone to publication and reporting bias: dramatic or successfully treated cases are more likely to be written up than fatal or diagnostically uncertain episodes, and the true denominator of patients at risk after RYGB or OAGB is unknown. Our review was restricted to English-language, full-text publications and may have missed relevant cases reported in other languages or formats. The small sample size precludes any meaningful comparison of perforation risk between OAGB and RYGB or any robust assessment of the independent contribution of specific risk factors such as NSAIDs, cholecystectomy, or bile reflux. Finally, our own case is subject to the same limitations as the other reports in the series: single-center, retrospective, and selected for publication because of its rarity, which should temper any attempt to generalize beyond carefully circumscribed clinical inferences.

### Clinical Implications and Future Directions

The practical implications of this review can be summarized as follows:

- Clinicians should maintain a high index of suspicion for excluded-stomach pathology in post-RYGB or post-OAGB patients presenting with persistent abdominal pain, systemic inflammatory response, and free intraperitoneal fluid, even in the absence of pneumoperitoneum or a clearly localized defect. Case series of acute gastric remnant complications after RYGB have shown that CT may demonstrate free fluid or remnant distension without visible free air, and that definitive diagnosis is often established only at laparoscopy [6, 7].
- Early diagnostic laparoscopy should be considered when clinical deterioration persists despite initial resuscitation and non-diagnostic imaging. In this setting, repeated imaging or prolonged observation may delay source control. This approach is consistent with current recommendations on the management of acute abdomen after MBS, which support timely minimally invasive exploration when alternative diagnoses have been reasonably excluded [49].
- Organ-preserving surgery was commonly reported and appeared feasible in most reported cases. Primary repair with omentoplasty or limited remnant resection when tissue quality is poor was frequently used for source control in the available reports. Routine completion gastrectomy is not supported by the available case-based evidence as a prophylactic or default emergency strategy and should be reserved for selected scenarios, such as diffuse non-reconstructable disease or suspected malignancy, in line with existing discussions on remnant gastrectomy for intractable ulcer disease or cancer risk [37].

Future reports and registry datasets should systematically document remnant-related complications, cholecystectomy status, bile reflux assessment, suspected mechanism, operative findings, histology, and long-term outcomes after RYGB and OAGB.

## Conclusion

Gastric remnant and duodenal perforation after RYGB or OAGB is a rare but serious complication that can occur from the early postoperative period to many years after MBS. Because the available evidence consists solely of case reports and small case series, definitive conclusions cannot be drawn regarding incidence, comparative procedural risk, or causative mechanisms. The available case-based evidence suggests that many reported perforations are ulcer-related, but the underlying mechanisms remain uncertain and are likely multifactorial. Persistent remnant activity, acid secretion, bile or pancreatic reflux, ischemia, medication exposure, and obstructive factors should be considered theoretical contributors rather than proven causes. Our late OAGB case illustrates the diagnostic ambiguity of excluded-stomach pathology.

Across published reports, minimally invasive, organ-preserving strategies, including primary repair with omentoplasty or limited remnant resection, were commonly reported and may achieve source control in selected patients. However, these observations remain limited by the case-based nature of the evidence. Current data do not support routine completion gastrectomy. MBS surgeons should maintain a low threshold for early diagnostic laparoscopy in bypassed patients with unexplained sepsis and free intraperitoneal fluid, even when CT does not show pneumoperitoneum or a clear perforation site, while recognizing that this recommendation is based on limited case-level evidence rather than comparative outcome data.

## Supplementary Information

Below is the link to the electronic supplementary material.

Supplementary Material 1

Supplementary Material 2

Supplementary Material 3

Supplementary Material 4

## Data Availability

All data generated or analyzed during this study are included in this article and its electronic supplementary materials. Additional clarifications are available from the corresponding author on reasonable request.

## Abbreviations

BMI: Body mass index
CT: Computed tomography
CRP: C–reactive protein
EGD: Esophagogastroduodenoscopy
ESM: Electronic Supplementary Material
H. pylori: Helicobacter pylori
ICU: Intensive care unit
IFSO: International Federation for the Surgery of Obesity and Metabolic Disorders
JBI: Joanna Briggs Institute
MBS: Metabolic and bariatric surgery
MGB: Mini–gastric bypass
NSAIDs: Non–steroidal anti–inflammatory drugs
OAGB: One–anastomosis gastric bypass
PPI: Proton–pump inhibitor
PRISMA: Preferred Reporting Items for Systematic Reviews and Meta–Analyses
RYGB: Roux–en–Y gastric bypass
