# Supplementary material for: Excluded-stomach Perforation after Roux-en-Y and One-anastomosis Gastric Bypass: A Systematic Review with Video-illustrated Case Report
Source: Obes Surg. 2026 May 20;36(6):3376–89. doi: 10.1007/s11695-026-08725-y (PMC13249912; doi:10.1007/s11695-026-08725-y)
Supplement: Supplementary file 1 — Supplementary Material 1 [file 11695_2026_8725_MOESM1_ESM.docx]

# Electronic Supplementary Material (ESM 1): Database Search Strategies (PubMed, Scopus, Web of Science)

## Database: PubMed (searched in January 2026)

**Search string:** ("one anastomosis gastric bypass" OR "mini gastric bypass" OR OAGB OR MGB OR "Roux-en-Y gastric bypass" OR RYGB OR "single anastomosis gastric bypass" OR "single-anastomosis gastric bypass" OR SAGB ) AND ("gastric remnant" OR "excluded stomach" OR "gastric stump" OR "bypassed stomach" OR "bypassed gastric remnant" OR "excluded gastric segment" OR "remnant stomach" OR "excluded gastric remnant" ) AND (perforation OR rupture OR ulcer* OR "peptic ulcer" OR "peptic ulcer disease" OR PUD OR "marginal ulcer" OR "anastomotic ulcer" OR "gastric perforation" OR "stomach perforation")

**Results:** 88 Results

## Database: Scopus (Elsevier)

**Search string:**

("one anastomosis gastric bypass" OR "mini gastric bypass" OR OAGB OR MGB OR "Roux-en-Y gastric bypass" OR RYGB OR "single anastomosis gastric bypass" OR "single-anastomosis gastric bypass" OR SAGB ) AND ("gastric remnant" OR "excluded stomach" OR "gastric stump" OR "bypassed stomach" OR "bypassed gastric remnant" OR "excluded gastric segment" OR "remnant stomach" OR "excluded gastric remnant" ) AND (perforation OR rupture OR ulcer* OR "peptic ulcer" OR "peptic ulcer disease" OR PUD OR "marginal ulcer" OR "anastomotic ulcer" OR "gastric perforation" OR "stomach perforation")

**Results:** 127 Results

## Database: Web of Science (Clarivate Analytics)

**Search string:**

("one anastomosis gastric bypass" OR "mini gastric bypass" OR OAGB OR MGB OR "Roux-en-Y gastric bypass" OR RYGB OR "single anastomosis gastric bypass" OR "single-anastomosis gastric bypass" OR SAGB ) AND ("gastric remnant" OR "excluded stomach" OR "gastric stump" OR "bypassed stomach" OR "bypassed gastric remnant" OR "excluded gastric segment" OR "remnant stomach" OR "excluded gastric remnant" ) AND (perforation OR rupture OR ulcer* OR "peptic ulcer" OR "peptic ulcer disease" OR PUD OR "marginal ulcer" OR "anastomotic ulcer" OR "gastric perforation" OR "stomach perforation")

**Results:** 97 Results

## Results total: 312
