# Supplementary material for: Excluded-stomach Perforation after Roux-en-Y and One-anastomosis Gastric Bypass: A Systematic Review with Video-illustrated Case Report
Source: Obes Surg. 2026 May 20;36(6):3376–89. doi: 10.1007/s11695-026-08725-y (PMC13249912; doi:10.1007/s11695-026-08725-y)
Supplement: Supplementary file 2 — Supplementary Material 2 [file 11695_2026_8725_MOESM2_ESM.docx]

# Electronic Supplementary Material (ESM 2): Risk of Bias—JBI Appraisal of Included Case Reports and Case Series

**Table S2.1:** JBI Case Report checklist (D1–D8: demographics; history/timeline; current condition; assessment methods/results; intervention; post-intervention condition; adverse events; takeaway/lessons) with study-level judgments.

| **Study** | D1: Patient's Demographics | D2: Patient's History | D3: Current Clinical Condition | D4: Assessment Method(s) | D5: Intervention Procedures | D6: Postintervention Clinical Conditions | D7: Adverse Events | D8: Takeaway Message |
| --- | --- | --- | --- | --- | --- | --- | --- | --- |
| **Tuero.C et al,2021** | Yes | Yes | Yes | Yes | Yes | Yes | Yes | Yes |
| **AlZarooni.N et al,2020** | Yes | Yes | Yes | Yes | Yes | Yes | Yes | Yes |
| **Ebrahimi.R et al,2019** | Yes | Yes | Yes | Yes | Yes | Yes | Yes | Yes |
| **Suri.K et al,2019** | Yes | Yes | Yes | Yes | Yes | Yes | Yes | Yes |
| **Munhoz.A et al , 2024** | Yes | Yes | Yes | Yes | Yes | Yes | Yes | Yes |
| **Dai.L et al,2018** | Yes | Yes | Yes | Yes | Yes | Yes | Yes | Yes |
| **Marquez.B et al,2023** | Yes | Yes | Yes | Yes | Yes | Yes | Yes | Yes |
| **Gnägi.C et al,2021** | Yes | Yes | Yes | Yes | Yes | Yes | Yes | Yes |
| **Laverty.R et al,2021** | Yes | Yes | Yes | Yes | Yes | Yes | Yes | Yes |
| **Papasavas.P et al,2003** | Yes | Yes | Yes | Yes | Yes | Yes | Yes | Yes |
| **Ovaere.S et al,2016** | Yes | Yes | Yes | Yes | Yes | Yes | Yes | Yes |
| **Shahabi.S et al,2025** | Yes | Yes | Yes | Yes | Yes | Yes | Yes | Yes |
| **Williams et al, 2024** | Yes | Yes | Yes | Yes | Yes | Yes | Yes | Yes |
| **Pereira et al, 2025** | Yes | Yes | Yes | Yes | Yes | Yes | Yes | Yes |
| **Aftab et al, 2025** | Yes | Yes | Yes | Yes | Yes | Yes | Yes | Yes |
| **Ay et al, 2022** | Yes | Yes | Yes | Yes | Yes | Yes | Unclear | Yes |
| **Gonçalves et al, 2025** | Yes | Yes | Yes | Yes | Yes | Yes | Yes | Yes |
| **Mittermair et al, 2007** | Yes | Yes | Yes | Yes | Yes | Yes | Yes | Yes |
| **Gypen et al, 2008** | Yes | Yes | Yes | Yes | Yes | Yes | Yes | Yes |
| **Nowotny et al, 2020** | Yes | Yes | Yes | Yes | Yes | Yes | Yes | Yes |
| **Peetermans et al, 2019** | Yes | Yes | Yes | Yes | Yes | Yes | Yes | Yes |
| **Hughes et al, 2023** | Yes | Yes | Yes | Yes | Yes | Yes | Unclear | Yes |

**Table S2.2:** JBI Case Series checklist (D1–D10: inclusion criteria; reliable measurement; valid identification; consecutive inclusion; complete inclusion; demographics; clinical information; outcomes/follow-up; site/clinician; statistics) with study-level judgments.

| **Study** | D1: Inclusion criteria defined | D2: Condition measured reliably | D3: Valid identification methods | D4: Consecutive inclusion | D5: Complete inclusion | D6: Demographics reported | D7: Clinical info reported | D8: Outcomes reported | D9: Site & clinician reported | D10: Appropriate statistics |
| --- | --- | --- | --- | --- | --- | --- | --- | --- | --- | --- |
| **Iranmanesh.P et al,2020** | Yes | Yes | Yes | Yes | Yes | Yes | Yes | Yes | Yes | Yes |
| **Iskandar et al, 2015** | No | Yes | Yes | Unclear | Unclear | Yes | Yes | Unclear | Yes | N/A |
| **Pohl et al, 2018** | No | Yes | Yes | Unclear | Unclear | Yes | Yes | Yes | Yes | N/A |
| **Zagzag et al, 2018** | Yes | Yes | Yes | Unclear | Unclear | Yes | Yes | Yes | Yes | No |
