# Supplementary material for: Excluded-stomach Perforation after Roux-en-Y and One-anastomosis Gastric Bypass: A Systematic Review with Video-illustrated Case Report
Source: Obes Surg. 2026 May 20;36(6):3376–89. doi: 10.1007/s11695-026-08725-y (PMC13249912; doi:10.1007/s11695-026-08725-y)
Supplement: Supplementary file 3 — Supplementary Material 3 [file 11695_2026_8725_MOESM3_ESM.docx]

# Electronic Supplementary Material (ESM 3): Extended Results—Time-to-Perforation and Etiology/Anatomical Site after RYGB and OAGB


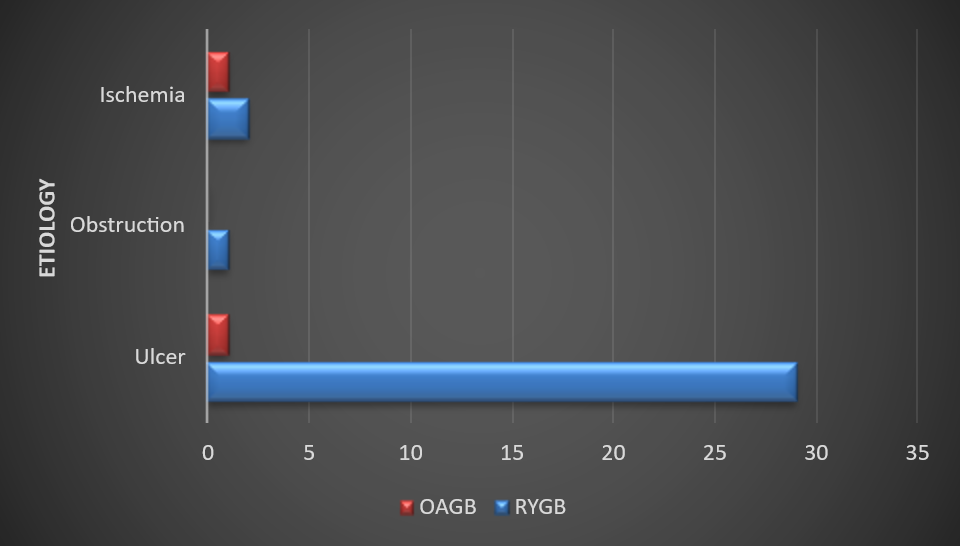


**Figure S3.1:** Time from index bypass to perforation showing the distribution in months overall and, where counts permit, stratified by procedure (RYGB vs OAGB); early (<30 days), intermediate (1–24 months), and late (>24 months) categories are indicated. Medians (IQR) and ranges are reported in the text/tables.


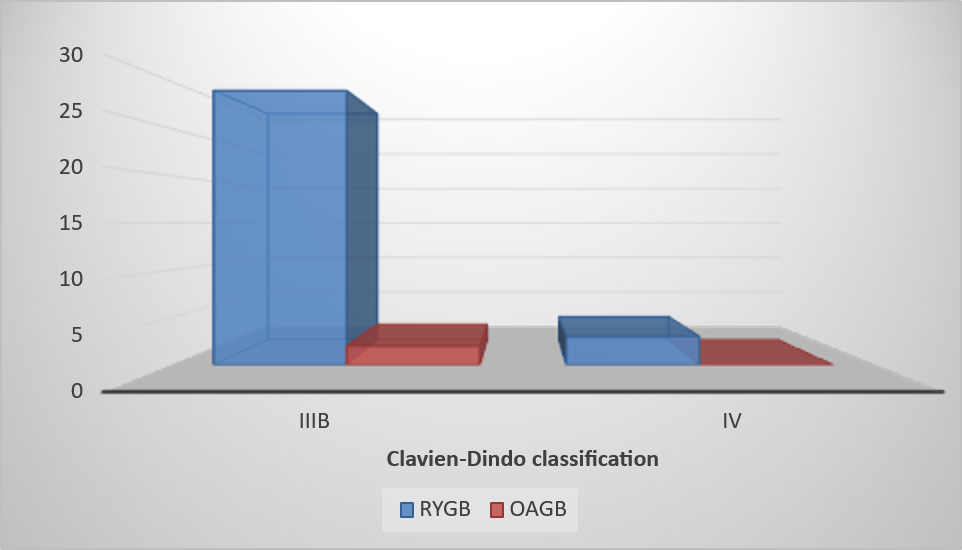


**Figure S3.2:** Etiology and anatomical site by procedure showing stacked distributions of mechanisms (ulcerative, ischemic, mechanical/iatrogenic, reflux-related, indeterminate) and predominant sites (pre-pyloric/pylorus, duodenum, other remnant) for RYGB and OAGB. Denominators vary by variable due to missing data; percentages are calculated on available cases. Abbreviations: RYGB, Roux-en-Y gastric bypass; OAGB, one-anastomosis gastric bypass.
